# Supplementary material for: A 16-week randomized controlled trial of a fish oil and whey protein-derived supplement to improve physical performance in older adults losing autonomy—A pilot study
Source: PLoS One. 2021 Aug 23;16(8):e0256386. doi: 10.1371/journal.pone.0256386 (PMC8382183; doi:10.1371/journal.pone.0256386)
Supplement: S1 Table — PL, phospholipid; Week 0: CTR n = 6/6, EXP n = 5/5; Week 8: CTR n = 6/6, EXP n = 5/5; Week 16: CTR n = 6/6; EXP n = 4/5. (DOCX) [file pone.0256386.s001.docx]

**S1 Table. Proportions of fatty acids in plasma phospholipids of participants by group**

|  | **CTR** | | | **EXP** | | |
| --- | --- | --- | --- | --- | --- | --- |
| Fatty acid (%) | **Week 0** | **Week 8** | **Week 16** | **Week 0** | **Week 8** | **Week 16** |
| C16:0 | 31.3 (28.9, 32.0) | 30.4 (27.8, 32.1) | 31.0 (28.7, 33.9) | 29.0 (28.4, 31.1) | 31.8 (28.4, 33.3) | 31.0 (30.4, 31.7) |
| C18:0 | 15.1 (14.0, 15.9) | 15.2 (14.5, 16.3) | 15.3 (12.6, 17.3) | 15.2 (14.0, 15.8) | 15.1 (14.6, 16.9) | 15.1 (14.7, 15.6) |
| C18:1n-9 | 10.0 (9.4, 13.1) | 11.6 (9.4, 12.9) | 10.9 (10.1, 12.1) | 11.7 (9.1, 15.0) | 9.4 (7.8, 12.7) | 9.9 (8.7, 11.8) |
| C18:2 n-6 | 18.5 (17.1, 23.1) | 18.4 (15.5, 23.4) | 18.2 (16.1, 19.6) | 18.3 (14.8, 23.3) | 14.6 (13.5, 19.4) | 18.5 (17.6, 19.7) |
| C18:3n-3 | 0.3 (0.1, 0.4) | 0.3 (0.2, 0.6) | 0.3 (0.3, 0.5) | 0.3 (0.2, 0.6) | 0.3 (0.2, 0.4) | 0.4 (0.3, 0.5) |
| C20:3n-3 | 0.1 (0.0, 0.9) | 0.1 (0.0, 0.5) | 0.1 (0.0, 0.3) | 0.1 (0.0, 0.1) | 0.1 (0.0,0.3) | 0.2 (0.0, 0.2) |
| C20:4 n-6 | 9.3 (6.9, 12.3) | 9.0 (6.8, 12.1) | 9.7 (8.6, 11.1) | 8.4 (6.9, 15.0) | 7.1 (5.6, 8.4) | 6.0 (5.4, 6.6) |
| C20:5n-3 | 0.8 (0.5, 2.1) | 1.2 (0.8, 1.8) | 1.0 (0.5, 1.3) | 1.1 (0.5, 4.3) | 3.3 (1.6, 6.6) | 2.4 (1.8, 3.6) |
| C22:5n-3 | 0.7 (0.5, 1.0) | 0.8 (0.7, 0.9) | 0.8 (0.6, 1.0) | 0.8 (0.6, 0.9) | 1.1 (0.9, 1.3) | 1.0 (0.7, 1.1) |
| C22:6n-3 | 2.2 (1.6, 3.4) | 2.3 (1.8, 3.2) | 2.4 (1.7, 2.9) | 3.0 (1.4, 4.2) | 4.4 (3.3, 7.1) | 4.0 (2.9, 4.7) |
| Total PUFA n-3 | 4.5 (3.4, 6.2) | 5.2 (4.0, 5.5) | 4.5 (3.7, 5.7) | 5.3 (3.7, 9.2 | 9.0 (6.4, 14.5) | 7.4 (6.6, 9.9) |
| Total PL fatty acids, ug/mL plasma | 351.2 (269.3, 805.5) | 383.8 (282.7, 967.7) | 543.7 (344.7, 723.9) | 555.7 (473.9, 702.9) | 499.2 (343.6, 653.1) | 367.9 (314.1, 545.6) |

Values are medians (range). PL, phospholipid; Week 0: CTR *n*=6/6, EXP *n*=5/5; Week 8: CTR *n*=6/6, EXP *n*=5/5; Week 16: CTR *n*=6/6; EXP *n*=4/5.
