# Supplementary material for: A 16-week randomized controlled trial of a fish oil and whey protein-derived supplement to improve physical performance in older adults losing autonomy—A pilot study
Source: PLoS One. 2021 Aug 23;16(8):e0256386. doi: 10.1371/journal.pone.0256386 (PMC8382183; doi:10.1371/journal.pone.0256386)
Supplement: S2 Table — Week 0: CTR n = 5/6, EXP n = 5/5; Week 8: CTR n = 5/6, EXP n = 4/5; Week 16: CTR n = 5/6; EXP n = 3/5. Daily average dietary intake without accounting for the supplement. M, men; W, women. (DOCX) [file pone.0256386.s002.docx]

**S2 Table. Dietary intake of participants by group**

|  | **CTR** | | | **EXP** | | |
| --- | --- | --- | --- | --- | --- | --- |
|  | **Week 0** | **Week 1** | **Week 2** | **Week 0** | **Week 1** | **Week 2** |
| Energy, M/W, kcal | 2354 / 1859 (944, 2669) | 1877 / 1644 (1034, 1731) | 1531 / 1357 (771, 2672) | 2250 / 1452 (1156, 2590) | n/a/1374 (814, 1730) | 2550 / 1545 (1257, 1805) |
| Protein, g/kg | 1.3 (0.7, 1.4) | 1.1 (0.3, 1.4) | 1.1 (0.3, 1.2) | 1.2 (0.9, 1.4) | 1.1 (0.4, 1.2) | 1.2 (0.8, 1.4) |
| Carbohydrates, g | 265 (106, 320) | 179 (144, 247) | 179 (120, 317) | 169 (105, 311) | 180 (108, 226) | 217 (134, 363) |
| Fat, g | 88 (31, 136) | 64 (29, 101) | 63 (21, 127) | 68 (50, 80) | 36 (31, 65) | 63 (45, 82) |
| Vitamin D, IU | 78 (13, 135) | 70 (25, 229) | 74 (46, 114) | 164 (63, 369) | 152 (32, 309) | 146 (97, 210) |
| DHA, g | 0.01 (0.00, 0.43) | 0.04 (0.00, 0.70) | 0.05 (0.02, 0.41) | 0.04 (0.02, 0.36) | 0.20 (0.01, 0.83) | 0.03 (0.02, 0.56) |
| EPA, g | 0.00 (0.00, 0.31) | 0.02 (0.00, 0.23) | 0.01 (0.00, 0.27) | 0.01 (0.00, 0.17) | 0.08 (0.00,0.40) | 0.02 (0.00, 0.27) |
| ALA, g | 3.14 (0.81, 4.31) | 1.57 (0.22, 2.38) | 0.60 (0.37, 6.57) | 1.03 (0.56, 1.63) | 0.77 (0.57, 0.89) | 1.39 (0.27, 1.95) |

Values are medians (range). Week 0: CTR *n*=5/6, EXP *n*=5/5; Week 8 : CTR *n*=5/6, EXP *n*=4/5; Week 16 : CTR *n*=5/6; EXP *n*=3/5

Daily average dietary intake without accounting for the supplement.

M, men; W, women.
