# Supplementary material for: A 16-week randomized controlled trial of a fish oil and whey protein-derived supplement to improve physical performance in older adults losing autonomy—A pilot study
Source: PLoS One. 2021 Aug 23;16(8):e0256386. doi: 10.1371/journal.pone.0256386 (PMC8382183; doi:10.1371/journal.pone.0256386)
Supplement: S3 Table — These numbers are applicable for participants weighing 65–75 kg. A factor of 0.8 and 1.2 needs to be applied to the total protein and amino acid profile (except for leucine) to obtain the actual amount consumed for those weighing <65 kg and >75 kg respectively. All amino acids are “L” isomers. *Each participant in the experimental group was given supplements containing both whey protein and leucine powder. Participants received 6 g of leucine powder independently of their weight. Thus, the total amount of leucine per day for participants weighing <65 kg was 10.5 g, and 12.76 g for those weighing >75 kg. (DOCX) [file pone.0256386.s003.docx]

**S3 Table. Nutritional composition of the multi-nutrient supplement and placebo for a one-day provision**

| Component | EXP | CTR |
| --- | --- | --- |
| Energy (kcal) | 240 | 290 |
| Carbohydrate (g) | 0 | 58 |
| Total protein (g) | 51 | 0 |
| Amino acid profile (g)   - Leucine* - Isoleucine - Valine - Histidine - Lysine - Methionine - Phenylalanine - Threonine - Tryptophan - Alanine - Arginine - Aspartic acid - Cysteine - Glutamic acid - Glycine - Proline - Serine - Tyrosine | 11.63  3.38  3.15  0.90  0.72  0.63  1.58  1.13  0.90  1.13  1.35  1.49  0.99  14.40  1.80  2.48  2.03  1.35 | -  -  -  -  -  -  -  -  -  -  -  -  -  -  -  -  -  - |
| Fat (g)   - Omega 3 (g) - Eicosapentaenoic acid (EPA) (g) - Docosahexaenoic acid (DHA) (g) | 4.57  1.875  1.125  0.750 | 6.8  0.075  -  - |
| Vitamin D (IU) | 1583 | 0 |

These numbers are applicable for participants weighing 65-75 kg. A factor of 0.8 and 1.2

needs to be applied to the total protein and amino acid profile (except for leucine) to obtain

the actual amount consumed for those weighing <65 kg and >75 kg respectively. All amino

acids are “L” isomers.

*Each participant in the experimental group was given supplements containing both whey

protein and leucine powder. Participants received 6 g of leucine powder independently of

their weight. Thus, the total amount of leucine per day for participants weighing <65 kg was

10.5 g, and 12.76 g for those weighing >75 kg.
