# Supplementary material for: A 16-week randomized controlled trial of a fish oil and whey protein-derived supplement to improve physical performance in older adults losing autonomy—A pilot study
Source: PLoS One. 2021 Aug 23;16(8):e0256386. doi: 10.1371/journal.pone.0256386 (PMC8382183; doi:10.1371/journal.pone.0256386)
Supplement: S1 File — (PDF) [file pone.0256386.s005.pdf]

Study protocol

REB 15-633-MUHC

Nutritional supplement to improve physical performance in MUHC-Geriatric Day Hospital participants: a pilot study

Principal Investigator:	Stéphanie Chevalier, PhD
Co-Investigator:	José A. Morais, MD
Study Coordinator:	Marie Lamarche, BSc
Graduate Student:	Anne-Julie Tessier, RD

Funding:

MUHC-Geriatric Medicine (Helen McCall Hutchison Scholarship Award 2015-2016)

Réseau québécois de la recherche sur le vieillissement of the Fonds de Recherche du Québec – Santé (FRQS)

BACKGROUND

Malnutrition is highly prevalent in the elderly population, ranging from 5-10% in community-dwelling individuals to more than 50% in hospitalized patients and those in rehabilitation and long-term care [1,2]. Our prior study (funded by the Helen McCall Hutchison Foundation) of frail elderly patients enrolled in the MUHC-Geriatric Day Hospital (GDH) rehabilitation program revealed that 6% of new attendees were frankly malnourished, an additional 13% were moderately malnourished and overall, 53% were at risk of malnutrition [3]. Malnutrition was associated with lesser muscle and fat mass, and handgrip strength, a measure of muscle strength. Scores of malnutrition (from the Mini-Nutritional Assessment, MNA) correlated with gait speed, a strong indicator of physical performance, mobility and even morbidity[4,5]. These findings justify testing a nutritional intervention aiming at improving nutritional status and physical performance of the GDH participants.

The comprehensive GDH program includes individualized physiotherapy, occupational health, nursing and medical care for rehabilitation of patients with loss of autonomy after hospitalization, falls, or rapid decline in their ability to perform activities of daily living. Unfortunately, no dietetic counseling is offered as part of the program. Therefore, for an eventual implementation of the program, our proposed intervention has to be pragmatic, feasible and targeted towards the main nutrients known to affect physical functioning, namely protein and vitamin D and possibly omega-3 fatty acids.

Dietary Protein Recommendations and Supplements. The official Recommended Daily Allowance (RDA) of protein for the elderly is currently actively debated and the suggestion to revise it upward is endorsed by many experts [6-8]. All acknowledge that the RDA should not merely cover the minimal protein needs to avoid deficiency but rather, the amount to minimize lean tissue loss, sustain optimal health and maintain an active living. In the Health, Aging and Body Composition study including 2066 men and women of 70-79 years, the loss of total lean and appendicular muscle mass was less in the highest quintile of energy-adjusted protein intake of 1.2 g/kg/d than in the lowest quintile of 0.8 g/kg/d[9]. Likewise, analysis of a subset of the Women's Health Initiative study (n=24,417 women, 65-79 years) determined a 32% lower risk of developing frailty over 3 years associated with a 20% increase in calibrated protein intake (as % of energy)[10]. From these and other studies, a protein intake of at least 1.2 g/kg/day should be advised [6,11].

The positive impact of protein supplementation on increasing gains in muscle mass and strength during prolonged resistance-type exercise is well demonstrated in young and older adults [12]. However, the role of protein supplement alone, in persons who cannot or do not want to engage in such exercise is more mitigated [13,14] and may largely depend on protein quality, dose and the timing of ingestion. Dietary protein contain non-essential and essential amino acids (AA), the latter recognized to be responsible for stimulating muscle protein synthesis and the post-prandial anabolic response [15]. Leucine is the most potent amino acid capable of stimulating protein synthesis through signaling to the intracellular nutrient-sensing mTORC1 resulting in increased mRNA translation and protein elongation [16]. We and others have proposed that a significant increase of circulating leucine is necessary to illicit anabolic effects in

muscle, the so-called anabolic threshold, which is elevated in older and particularly frail persons due to associated conditions such as inactivity, insulin resistance and inflammation [17,18].

Increasing protein intake from dietary sources such as meat, fish, dairy and legumes, in frail elderly persons is a challenge for the majority who have decreased appetite, and for many with mastication and digestibility difficulties, and budget limitations. Because large intakes of protein increase satiety and may induce a compensatory decrease in voluntary food intake [14], it is important to propose supplements of high-quality protein with fewer calories. Whey protein is naturally rich in essential AA, particularly leucine. It is palatable, easy to digest and dissolvable such that it may be provided as a small volume drink, which should not displace usual food intake. Whey protein supplements provided as 2 doses of 20 g per day were tested in mobility-limited elderly with resistance exercise training during 6 months [19]. Significant improvement in muscle mass and strength, and stair-climbing performance were reported due to exercise but only trends for additional benefits of the whey protein supplement were observed. The dose of 20 g per meal occasion may not have been sufficient to reach the anabolic threshold and, supplements were consumed after breakfast and supper. Our last year study, founded by the Helen McCall Hutchison foundation, revealed that a leucine supplement ingested before meals as opposed to within meals generated greater circulating leucine concentrations (both peak and area under the curve). Thus, timing of supplement ingestion should also be considered when designing an intervention to promote muscle protein anabolism.

A recent randomized placebo-controlled trial tested a whey protein supplement of two doses of 20 g enriched with 3 g leucine and 800 UI vitamin D, in sarcopenic, independently living older adults with mobility limitations [20]. Supplements were ingested before breakfast and lunch during 13 weeks. The intervention resulted in more gain in muscle mass and improved chair-stand test, an indicator of muscle strength and power, compared to placebo. No difference in handgrip strength and global score for physical performance were seen. Authors argued that the dose of whey protein may not have been sufficient to improve all aspects of physical performance.

Vitamin D and muscle health. Vitamin D deficiency is highly prevalent in older adults[21] . Investigations on the role of vitamin D and physical performance have yielded mixed results. Many studies, but not all, have reported positive associations between serum vitamin D levels and physical performance [22]. The majority of supplementation studies showed positive effects on balance, gait speed and muscle strength, but again, some did not. From a meta-analysis of RCTs, high-dose vitamin D supplements (700-1000 IU), raising serum levels ≥60 nmol/L, reduced the incidence of falls by 23%, but the low-dose supplements had no effect on falls [23]. It appears that normalizing serum vitamin D levels to sufficiency (50-80 nmol/L) in deficient individuals is desirable to optimize physical performance and high-dose supplements may provide further gains, perhaps additional to those resulting from whey protein and leucine supplement.

Omega-3 fatty acids. Long-chain omega-3 fatty acids (n-3 FAs) are essential in the diet and play important roles in organ development and function, including the brain and the heart. Notably, the marine-derived eicosapentaenoic acid (EPA) and docosahexaenoic acid (DHA) have well-known immune-modulating and anti-inflammatory properties and reduce the risk of coronary heart disease and stroke [24,25] and overall mortality in older adults [26]. Interestingly, recent studies have also revealed a role of n-3 FAs in bone [27] and muscle health and function [28-33]. Supplementation studies in humans have shown that n-3 FAs increased the postprandial stimulation of muscle protein synthesis and resulted in increased muscle mass and strength after 6 month-supplementation, in older adults [28], and enabled the maintenance of muscle mass of lung cancer patients during chemotherapy [29]. In an Islandic population-based study, higher plasma phospholipid polyunsaturated FAs were cross-sectionally associated with greater muscle size and leg strength, but not with changes over 5 years [30]. However, increased intake of total n-3 FAs and DHA were associated with lower risk of mobility disability in older women [31]. As well, in the InChianti prospective study, baseline plasma n-3 FA levels were inversely related with the risk of developing impaired physical performance but did not affect gait speed [32]. As with vitamin D, doses may be important. Indeed, 6 month-supplementation of 2 g DHA /day resulted in improvement in walking speed compared to placebo, in postmenopausal women [33].

Based on the above knowledge, we are aiming to provide a combined supplement consisting of whey protein isolate supplement given in 2 daily doses added with 3 g of leucine, before breakfast and before bedtime. Protein doses of 20 g, 25 g or 30 g of powder will be given according to body weight category. In addition, a supplement of 1.9 g of fish oil (long-chain omega-3 fatty acids EPA and DHA) containing 1500 IU vitamin D will be ingested once daily. Dosages are based on data showing elevating plasma phospholipids EPA + DHA to ≥4% of total FAs and 25(OH)D levels of ≥80 nmol/L, while being safe.

Rationale

Given the high prevalence of malnutrition of the GDH participants and its impact on poor physical performance, providing a nutritional supplement to increase protein, leucine, vitamin D and omega-3 FA intake should complement and optimize the rehabilitation interventions in improving muscle mass, strength and physical performance.

Objectives

Implementing the proposed nutritional supplement in the routine care of GDH participants will:
1. test the feasibility, acceptance and compliance to the supplement and its safety
2. provide pilot data to test the superiority of the supplement versus an isocaloric placebo on measures of muscle mass, strength, physical performance, and mobility.

Methods

Participants. All newly admitted patients, men and women, referred to the GDH program (of the MGH) who meet eligibility criteria will be invited to participate. Participants must be able to understand study requirements (Mini Mental State Exam, MMSE >22/30) and to read and speak English or French. Participants with the following criteria: BMI >35 kg/m^2, presence of kidney (GFR <30 mL/min/SA), liver or heart failure, stroke in the last 6 months (unless totally recovered), Parkinson's disease, severe neurologic conditions, active malignancies, acute inflammation (CRP >10 mg/L), known diagnostic of hyperparathyroidism, recent acute weight loss (>10% in 3 months, unless stabilized), allergy to milk and/or fish, long-term use of corticosteroids or anti-neoplastic medication, will be excluded from the study. All subjects taking vitamin D supplements ≥ 800 IU/d will be asked to reduce their dose to 400 IU/d. If vitamin D supplements were prescribed, the subjects' physician will be informed to change the current prescription to 400 IU/d for the study duration. As for subjects taking any type of omega-3 supplements, they will be asked to cease it for the study duration. All eligible subjects will be approached by the study coordinator and will be provided with an information brochure. Subjects interested in participating will be invited to review study procedures and sign the consent form.

Study design. This is a randomized, placebo-controlled, double-blinded pilot trial of two parallel arms. Participants will be randomly assigned to one of two groups: experimental (EXP) or control (CTR). Randomization will be achieved by computer-generated permuted block of 4 with stratification according to sex. Research staff and participants will be blinded to group allocations.

Intervention. Participants will be screened and baseline measures will be performed within their first 3 visits to the GDH. Upon randomization, EXP group will ingest a supplement of flavored whey protein isolate with added pure leucine (3 g) twice daily, before breakfast and before bedtime. Doses are adjusted per body weight as follows: 20 g, 25 g or 30 g per category of <65 kg, 65-75 kg and >75 kg of body weight respectively (see Appendix Table 2 for nutritional details). Supplements will be offered during 8 weeks (average time of GDH program attendance) and for the following 8 weeks, at home or residence, for a total of 16 weeks. The CTR group will ingest an isocaloric placebo consisting of 30 g maltodextrin (a simple carbohydrate, providing 116 kcal), following the same schedule. Both products will be provided in pre-weighted doses in sterile containers; participants will be instructed to dilute and mix with 125 mL (1/2 cup) water to ingest. The fish oil + vitamin D supplement will be provided as a liquid oil (NutraSea Liquid® from Ascenta). Participants will receive a dosing cup, pre-marked to measure 7.5 mL, providing 1500 IU vitamin D3 + 1125 mg EPA + 750 mg DHA, to be ingested once daily. CTR participants will receive an isocaloric placebo oil (corn oil) to be ingested similarly. Oil supplements may be mixed with the protein or maltodextrin powders or ingested separately according to taste. Participants already taking vitamin D (at lower doses than indicated in exclusion criteria) or omega-3 FA supplements may take part in the study but will be asked to interrupt their supplements for the duration of the study. Compliance will be evaluated by twice-weekly monitoring during GDH visits and frequent phone calls during the

second half of study, by self-reporting in a logbook and return of all empty containers and extra oil; ingestion of 80% of provided doses will be required to remain in the study.

Outcomes: all outcomes (except body composition) will be measured at baseline (prior to intervention), 8 weeks (last program visit) and 16 weeks (returning visit).

Primary outcome measures

♦ Feasibility: recruitment rate (≥ 50%), adherence to assessment tests (≥ 80%), adherence to supplements (≥ 80%)

♦ Physical performance from validated tests[34]:

- 6-minute walk test: distance achieved at normal walking pace for 6 minutes, measured on a 30 m-course; gait speed is also measured during the first 4 m, in m/s[35]. This test assesses the submaximal level of functional capacity and is indicative of mobility limitations; a change of 20 m has clinical relevance[36].
- 30-second chair-stand: number of times capable to stand up completely and sit down within 30 seconds, without the aid of armrests (measured in number of chair-stands).
- Timed-Up-and-Go (TUG): time required to rise from a chair, walk 3 m, turn and come back to sit on the chair, (measured in seconds).

♦ Muscle strength:

- handgrip strength measured with hand-held dynamometer, measured seated, 3 measurements per hand, alternatively (measured in kg)
- Maximal leg strength (isometric knee extension peak torque) from a 60-degree angle using the Biodex System 4 Pro dynamometer on the dominant side (measured in N).

Secondary outcome measures

♦ Appendicular muscle mass index will be measured by dual energy X-ray absorptiometry (DXA) as the sum of arm and leg lean tissues (without bone mineral content) divided by height squared. This index defines sarcopenia[37]. The test will be performed at the Montreal General Hospital or at the MUHC-McDonnell Centre for Innovative Medicine (Glen site).

♦ Voluntary physical activity measured by accelerometry. Participants will wear an ActiGraph® GT3X accelerometer (small belt-held device) during 4 days for each measurement. Instructions will be given on how and when to wear the device. The activity counts, energy expenditure, steps count, activity/sedentary bouts will be collected. An activity logbook will be completed during the same days.

♦ Food intake will be estimated from 3-day food diary. Instructions will be given on how to record food intake with portion size estimation with measuring cups and spoons, by a registered dietitian (PI or graduate student). Nutrient analysis will be performed using the Food Processor software (ESHA®).

***Other measures and questionnaires*.**

♦ Blood sample (fasting): complete blood count, full biochemistry, renal profile, A_1C, glucose, insulin, TSH, albumin, liver enzymes, C-reactive protein, vitamin D 25(OH). (Most are routine tests at the GDH, others will be requested). Aliquots will be centrifuged and separated, and plasma will be kept at minus 80°C for later analysis of phospholipid fatty acid composition by gas chromatography (GC-MS) and amino acid profile by liquid chromatography-tandem mass spectrometry (LC-MS/MS).

♦ Anthropometric measurements: height, weight, waist and calf circumference according to standard procedures.

♦ Questionnaires: Mini-Nutritional Assessment- Short Form (MNA-SF): 6-item questionnaire for screening of malnutrition; health-related quality of life (EQ-5D). Scores of Mini-Mental State Examination, Montreal Cognitive Assessment (MoCA), and Barthel index done at the GDH will be collected.

STATISTICAL ANALYSIS

This is a pilot study designed to generate data on feasibility, acceptance and compliance to the supplement and study tests; it is not powered for identifying statistical differences in the main outcomes. The sample size required for the full study will be of approximately 160 participants (80 EXP, 80 CTR). This calculation is based on an expected difference of 1 second in reducing the chair-stand time between the 2 groups, acknowledging that the CTR will also gain from the rehab program, and from standard deviation from published studies [19,20]. This sample size should be enough to detect differences in handgrip and leg strength and in appendicular muscle mass accretion. Pilot data will therefore be crucial to confirm these calculations. We aim to recruit 40 participants to provide confidence intervals to determine the final RCT sample size.

ETHICAL CONSIDERATIONS

All participants will provide written informed consent. Participants of both arms will benefit from a full nutritional counseling by the PI or the graduate student involved in this study, to optimize their nutritional intake in addition to the standard of care of the GDH program. Those randomized to the EXP group might further benefit from the supplement, in improving muscle mass strength and physical function, according to our hypothesis.

Intervention products and doses: The whey protein isolate, leucine and maltodextrin (placebo) powders are commercially available for human use and safe to ingest at the proposed doses. All will be purchased from ProteinCo Canada, Cowansville, QC, which certifies a Pharmaceutical Grade Purity. These powders are fairly easy to dissolve in water with some mixing and have a sweet taste. The total amount of protein supplemented per day (36 g, 45 g or 54 g) will increase total daily protein intake to 1.2-1.5 g/kg/day, depending on current intake. This range is recommended by at least two international expert groups and recognized as safe for the older population [6,11]. This dose is however not recommended for persons with severe kidney diseases; therefore, those having such diagnosis or an estimated glomerular filtration rate (eGFR) below 30 mL/min/SA will not be eligible to participate [11]. The free leucine (3 g) added to the whey protein will total 6 g, twice per day = 12 g, which is below the tolerable upper intake level (UL) and safe limit in humans of >550 mg/kg/d, equivalent to 30-39 g/d depending on

weight[38]. The leucine purity, of 99%, has been confirmed by LC-MS/MS and will be assessed for new lots purchased.

Vitamin D: the daily doses of $\geq$ 1966.7 IU (1966.7 IU, 1983.3 IU or 2100 IU depending on the body weight) of vitamin D3 is desirable to augment and reach serum 25(OH)D of $\geq$80 nmol/L, based on several studies mostly on osteoporosis and fractures [20,23,39]; it is well below the tolerable upper intake level of 4000 IU/day (DRIs) for adults >70 years.

Omega-3 fatty acids: Typical side effects include fishy aftertaste and burping, but these possible effects will be minimized by the choice of fish oil supplement NutraSea (from Ascenta) that is fruit-flavored (grapefruit/tangerine or apple). There is no UL determined for *n*-3 FAs. However, the European Food Safety Authority has established that supplemental doses up to 5 g/day "do not raise safety concerns for adults"[40]. Our proposed daily dose of 2 g is less and has been used in several other studies in older adults, of longer duration[28].

Potential risks: as with any change in diet, dietary supplements may be associated with gastro-intestinal adaptations at the onset (e.g. constipation or softer and more frequent stools), which typically resolve with time. If persisting, these will be addressed by dietary recommendations from the PI or graduate student (both RDs) or prescription of laxatives (by the study MD).
The functional tests proposed are standard and specific to the geriatric population, will be performed by trained physiotherapists of the GDH and should cause no harm. Any serious and non-serious adverse event will be recorded.

Financial compensation: participants will not receive an honorarium for this study. Their transport or parking fees for the three assessment visits (baseline, 8 weeks and 16 weeks) at the Royal Victoria Hospital (Glen site) will be reimbursed.

SIGNIFICANCE

The combined whey protein, leucine, vitamin D and omega-3 FAs supplement was carefully designed with appropriate dosages and timing of ingestion to be both relevant to study outcomes and practical to ensure compliance. This pilot study is a natural extension of our previous studies. It will help define the feasibility of implementing the proposed nutritional intervention in the GDH program to eventually extend to a larger RCT rigorously assessing the potential benefits of the supplement in improving physical performance of GDH participants and gaining back some losses of autonomy.

References

1. Kaiser MJ, Bauer JM, Ramsch C, et al. Frequency of malnutrition in older adults: a multinational perspective using the mini nutritional assessment. *J Am Geriatr Soc.* 2010;58(9):1734-1738.
2. Chevalier S, Desjardins I, Mainville D. Dépistage de la dénutrition et impact d'une intervention nutritionnelle chez les personnes âgées en soins de longue durée. *Nutrition-science en evolution.* 2008;6((1)):17-20.
3. Chevalier S, Saoud F, Gray-Donald K, Morais JA. The physical functional capacity of frail elderly persons undergoing ambulatory rehabilitation is related to their nutritional status. *J Nutr Health Aging.* 2008;12(10):721-726.
4. Guralnik JM, Ferrucci L, Pieper CF, et al. Lower extremity function and subsequent disability: consistency across studies, predictive models, and value of gait speed alone compared with the short physical performance battery. *J Gerontol A Biol Sci Med Sci.* 2000;55(4):M221-231.
5. Cesari M, Kritchevsky SB, Newman AB, et al. Added value of physical performance measures in predicting adverse health-related events: results from the Health, Aging And Body Composition Study. *J Am Geriatr Soc.* 2009;57(2):251-259.
6. Volpi E, Campbell WW, Dwyer JT, et al. Is the optimal level of protein intake for older adults greater than the recommended dietary allowance? *J Gerontol A Biol Sci Med Sci.* 2013;68(6):677-681.
7. Tang M, McCabe GP, Elango R, Pencharz PB, Ball RO, Campbell WW. Assessment of protein requirement in octogenarian women with use of the indicator amino acid oxidation technique. *Am J Clin Nutr.* 2014;99(4):891-898.
8. Rafii M, Chapman K, Owens J, et al. Dietary protein requirement of female adults >65 years determined by the indicator amino acid oxidation technique is higher than current recommendations. *J Nutr.* 2015;145(1):18-24.
9. Houston DK, Nicklas BJ, Ding J, et al. Dietary protein intake is associated with lean mass change in older, community-dwelling adults: the Health, Aging, and Body Composition (Health ABC) Study. *Am J Clin Nutr.* 2008;87(1):150-155.
10. Beasley JM, LaCroix AZ, Neuhouser ML, et al. Protein intake and incident frailty in the Women's Health Initiative observational study. *J Am Geriatr Soc.* 2010;58(6):1063-1071.
11. Bauer J, Biolo G, Cederholm T, et al. Evidence-based recommendations for optimal dietary protein intake in older people: a position paper from the PROT-AGE Study Group. *J Am Med Dir Assoc.* 2013;14(8):542-559.
12. Cermak NM, Res PT, de Groot LC, Saris WH, van Loon LJ. Protein supplementation augments the adaptive response of skeletal muscle to resistance-type exercise training: a meta-analysis. *Am J Clin Nutr.* 2012;96(6):1454-1464.
13. Paddon-Jones D, Rasmussen BB. Dietary protein recommendations and the prevention of sarcopenia. *Curr Opin Clin Nutr Metab Care.* 2009;12(1):86-90.
14. Fiatarone MA, O'Neill EF, Ryan ND, et al. Exercise training and nutritional supplementation for physical frailty in very elderly people. *N Engl J Med.* 1994;330(25):1769-1775.

15. Volpi E, Kobayashi H, Sheffield-Moore M, Mittendorfer B, Wolfe RR. Essential amino acids are primarily responsible for the amino acid stimulation of muscle protein anabolism in healthy elderly adults. *Am J Clin Nutr.* 2003;78(2):250-258.
16. Dodd KM, Tee AR. Leucine and mTORC1: a complex relationship. *Am J Physiol Endocrinol Metab.* 2012;302(11):E1329-1342.
17. Dardevet D, Remond D, Peyron MA, Papet I, Savary-Auzeloux I, Mosoni L. Muscle wasting and resistance of muscle anabolism: the "anabolic threshold concept" for adapted nutritional strategies during sarcopenia. *ScientificWorldJournal.* 2012;2012:269531.
18. Boirie Y. Fighting sarcopenia in older frail subjects: protein fuel for strength, exercise for mass. *J Am Med Dir Assoc.* 2013;14(2):140-143.
19. Chale A, Cloutier GJ, Hau C, Phillips EM, Dallal GE, Fielding RA. Efficacy of whey protein supplementation on resistance exercise-induced changes in lean mass, muscle strength, and physical function in mobility-limited older adults. *J Gerontol A Biol Sci Med Sci.* 2013;68(6):682-690.
20. Bauer JM, Verlaan S, Bautmans I, et al. Effects of a Vitamin D and Leucine-Enriched Whey Protein Nutritional Supplement on Measures of Sarcopenia in Older Adults, the PROVIDE Study: A Randomized, Double-Blind, Placebo-Controlled Trial. *J Am Med Dir Assoc.* 2015;16(9):740-747.
21. Annweiler C, Kabeshova A, Legeay M, Fantino B, Beauchet O. Derivation and validation of a clinical diagnostic tool for the identification of older community-dwellers with hypovitaminosis D. *J Am Med Dir Assoc.* 2015;16(6):536 e538-519.
22. Annweiler C, Schott AM, Berrut G, Fantino B, Beauchet O. Vitamin D-related changes in physical performance: a systematic review. *J Nutr Health Aging.* 2009;13(10):893-898.
23. Bischoff-Ferrari HA, Dawson-Hughes B, Staehelin HB, et al. Fall prevention with supplemental and active forms of vitamin D: a meta-analysis of randomised controlled trials. *Bmj.* 2009;339:b3692.
24. He K, Rimm EB, Merchant A, et al. Fish consumption and risk of stroke in men. *JAMA.* 2002;288(24):3130-3136.
25. Bucher HC, Hengstler P, Schindler C, Meier G. N-3 polyunsaturated fatty acids in coronary heart disease: a meta-analysis of randomized controlled trials. *Am J Med.* 2002;112(4):298-304.
26. Mozaffarian D, Lemaitre RN, King IB, et al. Plasma phospholipid long-chain omega-3 fatty acids and total and cause-specific mortality in older adults: a cohort study. *Ann Intern Med.* 2013;158(7):515-525.
27. Harris TB, Song X, Reinders I, et al. Plasma phospholipid fatty acids and fish-oil consumption in relation to osteoporotic fracture risk in older adults: the Age, Gene/Environment Susceptibility Study. *Am J Clin Nutr.* 2015;101(5):947-955.
28. Smith GI, Atherton P, Reeds DN, et al. Dietary omega-3 fatty acid supplementation increases the rate of muscle protein synthesis in older adults: a randomized controlled trial. *Am J Clin Nutr.* 2011;93(2):402-412.

29. Murphy RA, Mourtzakis M, Chu QS, Baracos VE, Reiman T, Mazurak VC. Nutritional intervention with fish oil provides a benefit over standard of care for weight and skeletal muscle mass in patients with nonsmall cell lung cancer receiving chemotherapy. *Cancer.* 2011;117(8):1775-1782.
30. Reinders I, Song X, Visser M, et al. Plasma phospholipid PUFAs are associated with greater muscle and knee extension strength but not with changes in muscle parameters in older adults. *J Nutr.* 2015;145(1):105-112.
31. Reinders I, Murphy RA, Song X, et al. Polyunsaturated fatty acids in relation to incident mobility disability and decline in gait speed; the Age, Gene/Environment Susceptibility-Reykjavik Study. *Eur J Clin Nutr.* 2015;69(4):489-493.
32. Abbatecola AM, Cherubini A, Guralnik JM, et al. Plasma polyunsaturated fatty acids and age-related physical performance decline. *Rejuvenation Res.* 2009;12(1):25-32.
33. Hutchins-Wiese HL, Kleppinger A, Annis K, et al. The impact of supplemental n-3 long chain polyunsaturated fatty acids and dietary antioxidants on physical performance in postmenopausal women. *J Nutr Health Aging.* 2013;17(1):76-80.
34. Guralnik JM, Simonsick EM, Ferrucci L, et al. A short physical performance battery assessing lower extremity function: association with self-reported disability and prediction of mortality and nursing home admission. *J Gerontol.* 1994;49(2):M85-94.
35. ATS statement: guidelines for the six-minute walk test. *Am J Respir Crit Care Med.* 2002;166(1):111-117.
36. Perera S, Mody SH, Woodman RC, Studenski SA. Meaningful change and responsiveness in common physical performance measures in older adults. *J Am Geriatr Soc.* 2006;54(5):743-749.
37. Cruz-Jentoft AJ, Baeyens JP, Bauer JM, et al. Sarcopenia: European consensus on definition and diagnosis: Report of the European Working Group on Sarcopenia in Older People. *Age Ageing.* 2010;39(4):412-423.
38. Pencharz PB, Elango R, Ball RO. Determination of the tolerable upper intake level of leucine in adult men. *J Nutr.* 2012;142(12):2220S-2224S.
39. Bischoff-Ferrari HA, Dawson-Hughes B, Whiting SJ. Vitamin D supplementation and fracture risk. *Arch Intern Med.* 2011;171(3):265; author reply 265-266.
40. EFSA Panel on Dietetic Products NaA. Scientific Opinion on the tolerable upper intake level of eicosapentaeinoic acid (EPA), docosahexaenoic acid (DHA) and docosapentaenoic acid (DPA). *European Food Safety Authority Journal.* 2012;10(7):2815.

Appendix

Table 1. Information on Dietary Supplements to be used

Supplement and ingredients	Brand Name/Company	Health Canada NPN	Daily Dose ingested	UL
Experimental supplement				
Whey protein isolate (vanilla flavored)<br><br>- Protein (27g/30g dose)<br>- Vitamin D3 (100 IU/30g dose)	New Zealand Whey Protein Isolate/ The Protein Company ATW Inc.	80054732	2 x 20 g = 40 g<br>2 x 25 g = 50 g<br>2 x 30 g = 60 g<br><br>36 g<br>45 g<br>54 g<br><br>66.7 IU<br>83.3 IU<br>200 IU	N/A<br><br>4000 IU/d
Leucine	ProteinCo Leucine Powder/ The Protein Company ATW Inc.	80062837	2 x 3 g = 6 g	550 mg/kg/d
Fish oil containing vitamin D (flavored) :<br>- EPA<br>- DHA<br>- Vitamin D3	NutraSea +D/ Ascenta	80050462	7.5 mL oil =<br><br>1125 mg<br>750 mg<br>1500 IU	EPA + DHA : 5 g/d<br><br>4000 IU/d
Placebo supplement				
Maltodextrin	Maltodextrin Powder/ The Protein Company ATW Inc.	80054728	2 x 30 g= 60 g	N/A
Corn Oil		N/A	7.5 mL	N/A

UL: Upper Tolerable Level; EPA : eicosapentaenoic acid; DHA : docosahexaenoic acid; N/A: not applicable
