# Supplementary material for: A 16-week randomized controlled trial of a fish oil and whey protein-derived supplement to improve physical performance in older adults losing autonomy—A pilot study
Source: PLoS One. 2021 Aug 23;16(8):e0256386. doi: 10.1371/journal.pone.0256386 (PMC8382183; doi:10.1371/journal.pone.0256386)
Supplement: Attachment — Submitted filename: Response to Reviewers.docx [file pone.0256386.s006.docx]

RESPONSE TO REVIEWERS - PONE-D-20-25559-R1

SC: We wish to thank the reviewers once more for the time and efforts invested in reviewing our manuscript and for providing constructive comments and suggestions. We have addressed all questions and comments of reviewer 3 below, and made appropriate modifications to the manuscript.

Reviewers' comments:

Reviewer's Responses to Questions

**Comments to the Author**

1. If the authors have adequately addressed your comments raised in a previous round of review and you feel that this manuscript is now acceptable for publication, you may indicate that here to bypass the “Comments to the Author” section, enter your conflict of interest statement in the “Confidential to Editor” section, and submit your "Accept" recommendation.

Reviewer #1: All comments have been addressed

Reviewer #2: All comments have been addressed

Reviewer #3: (No Response)

2. Is the manuscript technically sound, and do the data support the conclusions?

The manuscript must describe a technically sound piece of scientific research with data that supports the conclusions. Experiments must have been conducted rigorously, with appropriate controls, replication, and sample sizes. The conclusions must be drawn appropriately based on the data presented.

Reviewer #1: (No Response)

Reviewer #2: Yes

Reviewer #3: No

3. Has the statistical analysis been performed appropriately and rigorously?

Reviewer #1: (No Response)

Reviewer #2: Yes

Reviewer #3: Yes

4. Have the authors made all data underlying the findings in their manuscript fully available?

The [PLOS Data policy](http://www.plosone.org/static/policies.action#sharing) requires authors to make all data underlying the findings described in their manuscript fully available without restriction, with rare exception (please refer to the Data Availability Statement in the manuscript PDF file). The data should be provided as part of the manuscript or its supporting information, or deposited to a public repository. For example, in addition to summary statistics, the data points behind means, medians and variance measures should be available. If there are restrictions on publicly sharing data—e.g. participant privacy or use of data from a third party—those must be specified.

Reviewer #1: (No Response)

Reviewer #2: Yes

Reviewer #3: No

5. Is the manuscript presented in an intelligible fashion and written in standard English?

PLOS ONE does not copyedit accepted manuscripts, so the language in submitted articles must be clear, correct, and unambiguous. Any typographical or grammatical errors should be corrected at revision, so please note any specific errors here.

Reviewer #1: (No Response)

Reviewer #2: Yes

Reviewer #3: Yes

6. Review Comments to the Author

Please use the space provided to explain your answers to the questions above. You may also include additional comments for the author, including concerns about dual publication, research ethics, or publication ethics. (Please upload your review as an attachment if it exceeds 20,000 characters)

Reviewer #1: (No Response)

Reviewer #2: (No Response)

Reviewer #3: This article is well written and attempts to address current gaps in the scientific literature related to frailty/sarcopenia/aging and a multi-nutrient supplement containing whey protein, n-3 PUFAs, and vitamin D. It is unfortunate this pilot study failed to recruit the minimal number of participants, resulting in the inability to identify significant findings.

1.) The concept of autonomy loss in aging is multifactorial and unclear. Is there a cut-off for low, moderate, or high autonomy? It seems as if your methodology measures frailty/sarcopenia/dynapenia. In addition, the introduction (line 30, 34, 41, 50) citations refer to a sarcopenia (reference 3, 8, 9, 10) and frailty (reference 4).

A more common identifiable term with reference values would be more useful when applying this work to future research. Assuming all participants do not fall under one category you may identify multiple conditions to monitor. Please choose a more replicable and meaningful term.

The referral of participants to the Geriatric Day Hospital rehabilitation program was based on subjective impression of loss of independence rather than objective measures, though these measures do exist. This explains why we measured and defined frailty, sarcopenia and dynapenia using published cut-offs, precisely to better characterize the heterogenous population under study. The vast majority (85%, all but 3 at baseline) had mobility limitations requiring a walking aid. When examining our data again to find a common feature, it is clear that participants had low functional capacity, as defined by a distance of 400 m or less during the 6-minute walk test, an endurance test (ATS Guidelines). There are no strictly -defined cut-offs but the 400 m threshold is often used to define low functional capacity according to normative data (Casanova et al. Eur Respir J 2011; 37:150-156). Only two participants had distances just above 400 m, at 413 and 415 m. Therefore, we revised our manuscript throughout to qualify our participants as having low functional capacity.

Nonetheless, your valuable comment on the imprecision of the term “loss of autonomy” led us change it
for “loss of independence” which, from a quick PubMed search, seems to be more commonly used to define the same concept. (We also realized that “loss of autonomy” might come from a literal translation from the French “*perte d’autonomie*” commonly used in the geriatric jargon). The term may better resonate to readers. We also added more explanation regarding the referral of participants to the program.

We use the appropriate term loss of independence and tried to clarify its meaning in the introduction (lines 36-40 and 60-64), methods (lines 71-74), results (lines 252-253) and discussion sections.

2.) The statistical analyses are basically confidence intervals and are displayed as medians and percentiles. I understand that this may be appropriate due to the extremely low sample size. However, the statements in the abstract are misleading. As statistical analyses were not conducted to differentiate significance from non-significance, stating total lean body mass increased and strength decreased in the EXP group is misleading and unclear. In addition, the EXP group had no change in appendicular lean body mass, compared to the CTR group. At minimum, the increase/decrease in LBM, ALM, and strength should be delineated as total change, rather than by “increased and decreased” similar to the other parameters delineated in the abstract.

Indeed, extensive statistical analysis would be inappropriate with such limited sample size. However, stating that LBM increased and that strength decreased in the EXP group is not misleading since the 95% CI of the change from baseline is all positive for LBM (+508.3 g, 95%CI: 142.3, 874.2) and all negative for strength (mean: -2.5 kg, 95%CI: -4.3, -0.6) (Figure 3 and page 17 lines 327-332). We acknowledge that the statement in the abstract may be unclear because it was incomplete: changes were not different between groups (as 95% CI of changes are overlapping). A statistical test (with a p value) is not necessary to perform to visually appreciate these findings in Figure 3.

Because of word limit in the abstract, that sentence was removed.

3.) The abstract does not indicate the number of participants in the CTR versus EXP group. Due to the imbalance and small sample size, this should be stated in the abstract.
Number of participants has been added to abstract: 6 CTR, 4 EXP.

4.) I disagree with the conclusion of a high supplement compliance rate. The n-3 PUFAs + Vitamin D oil supplement intake decreased from 86% at week 8 to 55% at week 16 in EXP (line 250). This is a major limitation and should not be described as a success. Decreased compliance may have contributed lack of change in EPA and DHA plasma phospholipids at week 16. If DHA and EPA significantly increased at week 8, but not week 16 is it even relevant? Were these participants losing motivation throughout the intervention, becoming frailer, or fatigued for reasons related and not related to your intervention? How can a gradual loss of compliance be mitigated in the future? These concepts need to be described in the limitation’s section of the manuscript. A high-compliance rate should be removed or modified in the abstract and conclusion.

We agree with this comment and have tempered the “successful” adherence to fish oil supplement at 16 weeks. Because of space limitation in the abstract, we have to pool results of compliance to powder (experimental + placebo) and oil (experimental + placebo) together but a clarification on the decline in adherence to fish oil is now part of the conclusion. DHA was still significantly increased at week 16 but not EPA. We believe that increases at 8 weeks that were not sustained over 16 weeks are relevant; they support acceptance of the fish oil supplement but highlight the need for mitigation strategies to sustain motivation over longer intervention periods. As suggested, a sentence was also added to the paragraph on limitations to propose potential mitigation strategies (p.24 lines 482-484) : “Maintaining high adherence to the fish oil supplement up to 16 weeks remains a challenge that could be addressed through other means of delivery, such as part of favorite foods, change of flavors and more intense follow-up”.

5.) Did you monitor sunlight exposure? If not, this should be added to the limitation’s sections. This may explain the decrease in the control group at week 16.
Sunlight exposure was not monitored but was likely very limited in both groups given the mobility limitations impeding walking outside, as supported by the low average step count (mean 1406 steps/day). For this reason, we consider the lack of sunlight exposure to have a trivial impact on serum 25(OH)D levels as compared to stopping habitual vitamin D supplements.

6.) Why did you choose the n-3 PUFA and vitamin D doses? You suggest this RCT provides the foundation to support the elaboration of a large-scale intervention. The doses chosen should be adequately justified/referenced in the manuscript.
The fish oil doses were chosen based on previous studies led by our co-investigator Vera Mazurak having reported maintenance or gain in muscle mass in cancer patients undergoing chemotherapy with ingestion of these doses over an average of 10 weeks (ref 17, Murphy et al. Cancer 2011).

The vitamin D dose of 1500 IU/day was chosen based on an effective dose of 800 IU/d associated with decreased risks of falls (ref 18, Bischoff-Ferrari et al. BMJ 2009), a safe upper tolerable level of 4000 IU/day and is commensurate with prescribed vitamin D supplements to adults of 65 years and older in Québec, of 10,000 IU per week. Please see additions on p.7, lines 139-144.

Minor:

Line 413: add space between younger and reference 39.
Done

Table 1. Baseline descriptive characteristics of participants- Units are missing from parameters including and following “Waist circumference”. Add the appropriate units.

Units have been added.
